# Beyond the gut: decoding the gut–immune–brain axis in health and disease

**DOI:** 10.1038/s41423-025-01333-3

**Published:** 2025-08-14

**Authors:** John Chulhoon Park, Leechung Chang, Ho-Keun Kwon, Sin-Hyeog Im

**Affiliations:** 1 Department of Life Sciences, POSTECH Biotech Center, Pohang University of Science and Technology (POSTECH), Pohang, Republic of Korea; 2 Department of Microbiology and Immunology, Yonsei University College of Medicine, Seoul, Republic of Korea; 3 Brain Korea 21 PLUS Project for Medical Sciences, Yonsei University College of Medicine, Seoul, Republic of Korea; 4 Institute for Immunology and Immunological Diseases, Yonsei University College of Medicine, Seoul, Republic of Korea; 5 Institute for Convergence Research and Education in Advanced Technology; Yonsei University, Seoul, Republic of Korea; 6 ImmmunoBiome Inc., Pohang, Republic of Korea

**Keywords:** Gut–immune–brain axis, Gut microbiota, Neuroimmunology, Barrier integrity, Neurological disorders, Neuroimmunology, Mucosal immunology, Predictive markers

## Abstract

Emerging research underscores the pivotal role of the gut–immune–brain axis, a dynamic bidirectional communication system involving intricate interactions between the gut microbiota, immune responses, and the central nervous system. Gut microbes and their metabolites have profound effects on immune and neurological homeostasis, influencing the development and function of multiple physiological systems. Disruption of the composition of the gut microbiota and barrier integrity has been implicated in various neurological and psychiatric disorders, including autism spectrum disorder, Alzheimer’s disease, Parkinson’s disease, depression, and anxiety. Most insights into these host–microbiota interactions come from preclinical models, revealing both the complexity and potential therapeutic opportunities of the gut–brain communication pathways. This review synthesizes the current understanding of these intricate interactions, exploring how microbiota-driven modulation of the gut and brain barriers, immune signaling, and neuronal pathways, such as those through the vagus nerve, contributes to health and disease. We further explore therapeutic implications, including personalized precision microbiota interventions, microbiome-derived biomarkers, and barrier-strengthening strategies. Advancing this field offers transformative potential for developing innovative, personalized therapies tailored to individual microbiomes and immune profiles, ultimately redefining clinical approaches to neurological and immune-mediated diseases.

## Introduction

The human body harbors a vast and diverse community of commensal microbes, with the gut microbiota playing a crucial role in regulating host physiology. Recent research has demonstrated that gut microbes not only influence metabolism and host immunity, but also modulate neurodevelopment and brain function [1, 2]. The concept of a gut‒brain axis has emerged as a major paradigm shift in neuroscience, revealing bidirectional communication pathways between the gastrointestinal tract and the central nervous system (CNS) [2]. While this axis was initially thought to rely on neural and endocrine signaling, accumulating evidence now suggests that the immune system is a critical intermediary in gut‒brain communication, forming what is increasingly recognized as the gut‒immune‒brain axis. The first evidence linking the gut microbiota to brain function came from studies in the early 21st century using germ-free (GF) mice. These studies revealed that the absence of the gut microbiota leads to alterations in stress responses, neurotransmitter levels, and neurodevelopment [3, 4]. The colonization of GF mice with specific microbiota restores normal levels of stress hormones and brain-derived neurotrophic factor (BDNF), highlighting the influence of microbes on neuronal function [5]. However, these early studies largely focused on direct neuronal interactions, leaving the potential role of the immune system largely unexplored.

Historically, the brain has been considered an immune-privileged organ that is shielded from peripheral immune interactions by the blood‒brain barrier (BBB) [6]. However, recent findings have challenged this notion, showing that immune cells actively infiltrate the brain and play essential roles in neurodevelopment, homeostasis, and disease [7, 8]. The discovery of tissue-resident T cells and border-associated macrophages in the CNS has further underscored the presence of a functional neuroimmune network [9, 10]. These findings, combined with evidence that systemic inflammation contributes to neurodegenerative and neuropsychiatric disorders, suggest that immune mechanisms are central to neural health and disease. Concurrently, research into gut microbiota–immune interactions has revealed intricate pathways by which microbial metabolites shape immune function [11]. The gut microbiota is essential for the development and regulation of both innate and adaptive immunity, producing bioactive metabolites such as short-chain fatty acids (SCFAs), tryptophan derivatives, and secondary bile acids, which modulate systemic inflammation and immune responses [12]. Importantly, these microbial signals have been shown to impact brain functions through both direct metabolic pathways and by shaping immune cell activity in the periphery, which in turn influences neuroinflammation [13].

Despite the increasing recognition of the gut‒immune‒brain axis, many mechanistic questions remain unresolved. The precise pathways through which gut-derived immune signals influence the CNS, the relative contributions of different immune populations, and the extent to which microbiota-targeted interventions can modulate brain function are still unclear. Recent studies suggest that gut microbiota dysbiosis may drive neuroinflammation in multiple neurological disorders, highlighting the translational relevance of this field [2].

**Fig. 1:**
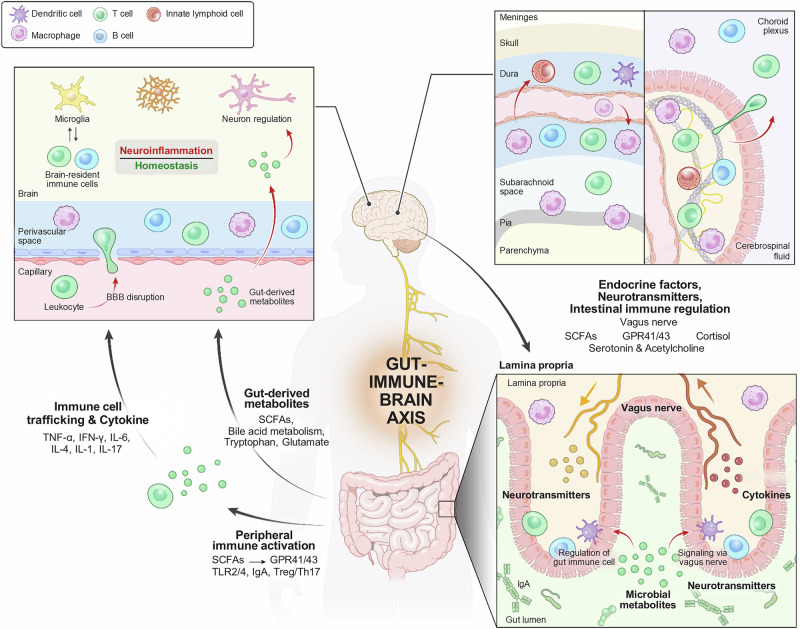
Schematic of the tridirectional gut‒immune‒brain axis. The gut harbors a complex microbiota capable of producing metabolites that regulate host physiology. These metabolites, such as SCFAs, can influence host immune maturation and the balance between pro- and anti-inflammatory responses. On the other hand, immune signals, such as IgA and the Treg/Th17 balance, can regulate microbial tolerance. Microbial metabolites can modulate neuroinflammation and homeostasis by directly affecting brain-resident immune cells and neurons and by eliciting peripheral immune responses that cascade into the brain. Owing to bidirectional crosstalk, the brain itself can modulate the gut microbiota through the vagus nerve and the HPA axis

In this review, we integrate findings from the gut microbiota, immunology, and neurobiology fields to elucidate the gut‒immune‒brain axis (Fig. 1). We first outline the fundamental principles of mucosal immunology and microbiota‒immune interactions, and then examine the roles of CNS‒resident and CNS‒infiltrating immune populations in brain function. Finally, we synthesize emerging evidence to propose a mechanistic framework for gut-derived immune modulation of the CNS and discuss its implications for neurological disorders. As this field continues to expand, a deeper understanding of these interactions will provide novel therapeutic avenues for neuroinflammatory and neuropsychiatric diseases.

## Gut–immune interactions

### Early-life development of the gut microbiota and host immunity

Early-life development presents a crucial window during which the commensal microbiota shapes the maturation and functional programming of the host immune system [14]. The first immune cells of the fetus arise from the yolk sac and include premacrophages from erythro-myeloid progenitors, mast cells, natural killer cells, and innate lymphoid cells [15–17]. Neuroimmune cells, such as microglia, can be found in fetal neural tissue as early as 4.5 gestational weeks [18]. Moreover, T-cell development occurs later in the fetal thymus after 6 gestational weeks, with memory phenotypes arising in the second trimester [19, 20]. The maternal microbiome undergoes significant changes throughout pregnancy, with notable enrichment of Proteobacteria and Actinobacteria, as well as overall lower richness toward the third trimester [21, 22]. The progressive shifts in the maternal microbiome correspond to changes in the metabolite pool and the maternal immune system [23, 24]. Notably, maternal microbiota-derived metabolites, including secondary bile acids, have been identified in fetal intestines and may shape the infant immune system [25]. Moreover, a study comparing the fetal metabolome of specific pathogen-free (SPF) and GF mice demonstrated drastic differences among metabolites known to harbor immunological potential [26].

The impact of the maternal microbiota on offspring is further compounded at birth, when neonatal mucosal surfaces are rapidly colonized by microbial genera such as *Lactobacillus, Staphylococcus*, and *Streptococcus* [27, 28]. Throughout infancy, the microbiota composition is dynamically shaped by dietary factors, antibiotic exposure, infections, and environmental stimuli [28]. For example, a large body of literature demonstrates the impact of breastfeeding on infant gut microbial compositions [29]. Moreover, breastfed infants have been reported to harbor greater frequencies of blood leukocytes [30]. The essential role of the gut microbiota in immune development is highlighted in GF mice, which exhibit significant reductions in immune cell populations, including macrophages, dendritic cells, neutrophils, T cells, and B cells, along with lower cytokine production [31, 32]. Mice treated with antibiotics early in life also retain microbial, immunological, and neurophysiological disruptions well into adolescence [33]. Notably, microbiota reintroduction into GF mice partially restores immune cell populations and function, depending on bacterial strain composition and the timing of colonization [34, 35]. With the transition into adolescence, the gut microbiota composition develops further, which may be in part due to changes in sex hormone levels [36]. Adolescents undergo immunological changes, including functional changes in glial cells and the BBB, although it is not clear whether these changes are tied to those in the gut microbiota [37]. Importantly, this evidence highlights the critical windows during which microbiota‒immune interactions take place and their potential implications for later life.

### Effects of the commensal microbiota on mucosal immunity

The mucosa-associated lymphoid tissue (MALT), which spans the oral, nasal, pulmonary, cutaneous, and gastrointestinal tracts, serves as a key interface for microbial‒immune interactions. Among these, the gastrointestinal mucosa harbors the most extensive immune network, continuously engaging with commensal microbes to regulate immune homeostasis [38]. In GF mice, the absence of commensal bacteria specifically leads to underdeveloped mucosal lymphoid structures, including Peyer’s patches and germinal centers, as well as reduced secretory immune factors [35, 39]. Microbiota-derived signals are essential for the differentiation and function of various immune cell populations. Microbial-derived peptides and polysaccharides promote the expansion of intestinal regulatory T cells (Tregs) and their production of anti-inflammatory IL-10, whereas segmented filamentous bacteria (SFB) drive Th (T helper) 17 differentiation and their production of proinflammatory IL-17 [40, 41]. Similarly, the composition of innate lymphoid cells (ILCs) is influenced by the microbiota, with *Clostridium rodentiae* enhancing ILC1 populations, *Helicobacter pylori* driving ILC2 expansion, and *Salmonella typhimurium* increasing ILC3 populations [42]. A critical mechanism through which the microbiota shapes mucosal immunity is IgA production, the most abundant antibody at mucosal surfaces [43, 44]. IgA not only neutralizes pathogens but also plays a key role in shaping the microbiota composition. Its production is regulated by T follicular helper (Tfh) cells, whose activity is modulated by specific commensal microbes, such as *Anaeroplasma* species [45]. T follicular regulatory cells further ensure IgA homeostasis, preventing excessive or nonspecific antibody responses while maintaining microbial equilibrium [46]. Thus, the gut microbiota and mucosal immune cells engage in a dynamic and reciprocal relationship in which microbial signals drive immune maturation, and in turn, immune responses shape the microbiota composition and function.

### Mechanisms of microbiota‒immune cell crosstalk

SCFAs, which are produced primarily by microbial fermentation of dietary fiber, are essential regulators of innate and adaptive immunity [47]. SCFAs interact with G protein-coupled receptors (GPRs), such as GPR41, GPR43, and GPR109A, suppressing NF-κB activation and thereby modulating inflammatory cytokine production [48–51]. Additionally, SCFAs act as histone deacetylase (HDAC) inhibitors to regulate T-cell differentiation, promoting Treg differentiation and influencing inflammatory responses [52]. In addition to metabolic signaling, microbial-associated molecular patterns (MAMPs) engage the immune system through recognition by toll-like receptors (TLRs) on antigen-presenting cells, lymphocytes, fibroblasts, and epithelial cells [53]. TLRs serve as key sensors in immune surveillance, detecting microbial components and initiating context-dependent immune responses. For example, TLR4 recognizes bacterial lipopolysaccharides (LPS) and activates the NF-κB and interferon pathways, driving proinflammatory cytokine production [54]. Moreover, TLR2, which responds to surface polysaccharides from probiotic bacteria, promotes anti-inflammatory pathways by inducing Tregs [40]. TLR2 signaling is also crucial for Tfh cell function, thereby influencing IgA-mediated microbiota regulation [55]. In this manner, the direct activation of different TLRs by their respective microbial components can elicit targeted immune responses to maintain homeostasis, clear pathogens, and serve as avenues for therapeutics.

### Effects of commensal microbes on systemic immunity

The gut microbiota influences not only mucosal immunity but also the development and regulation of systemic immune responses during both homeostasis and disease [56]. As in mucosal tissues, commensal microbes are essential for the proper maturation and function of systemic immune cells. Compared with their SPF counterparts, young GF mice exhibit reduced thymus sizes and decreased numbers of lymphocytes, indicating a critical role for the microbiota in conventional T-cell development [57, 58]. Gut microbiota-derived antigens are transported to the thymus via migratory dendritic cells, where they facilitate the expansion of antigen-specific T cells [59]. GF mice also exhibit impaired proliferation and TCR signaling in unconventional T cells, including mucosal-associated invariant T (MAIT) cells, highlighting the necessity of microbiota-derived metabolites for MAIT cell development [60]. Similarly, the expression of an autoimmune regulator (*Aire*) in thymic epithelial cells is necessary for the training and regulation of self-reactive T cells [61]. In GF mice, thymic epithelial cells exhibit reduced *Aire* expression, which can be restored by colonization with specific gram-positive bacteria, suggesting that microbial cues are required for proper T-cell clonal selection [61, 62]. These findings provide compelling evidence that the gut microbiota can drive systemic immune modulation, offering novel insights into host–microbiota crosstalk and potential therapeutic interventions.

### Key players in neuroimmune interactions during health

Although the CNS has long been considered an immune-privileged organ, recent studies have begun to highlight the pivotal role of the immune system in brain development, homeostasis, and disease. Immune cells of the CNS are primarily compartmentalized within the meninges, choroid plexus, and parenchyma, each with distinct structures and barriers that regulate molecular exchange and immune accessibility. The meninges, consisting of the dura, arachnoid, and pia mater, serve as immunological barriers encasing the brain and the spinal cord. The meningeal lymphatic system facilitates CNS immune surveillance by draining cerebrospinal fluid (CSF) and macromolecules, monitoring CNS-derived antigens to sustain homeostasis [63]. The dura mater, a highly vascularized layer with fenestrated capillaries, harbors border-associated macrophages (BAMs), T cells, B cells, and dendritic cells that detect and respond to peripheral immune signals. The leptomeninges (arachnoid and pia mater) house fewer immune cells but contain CSF and cytokines. Unlike the dura, leptomeningeal blood vessels lack fenestrations and are sealed by endothelial tight junctions [64]. The arachnoid mater is also reinforced by tight junctions and demarcates the dura from the subarachnoid space. Dural molecules can only enter the subarachnoid space through arachnoid cuff exit points, which are localized permeable regions [65]. The choroid plexus, located in the brain ventricles, serves as both a CSF producer and a critical neuroimmune interface. Like the dura, it is highly vascularized with fenestrated capillaries, permitting selective molecular exchange. It harbors macrophages, dendritic cells, and T cells, which contribute to CNS immune surveillance. The blood–CSF barrier in the choroid plexus controls molecular and immune cell entry into the CSF, ensuring CNS homeostasis [66]. These structures collectively establish a dynamic CNS immunological landscape in which immune cells interact with other brain cells to regulate CNS functions. This section examines key neuroimmune players, including classical glial cells (microglia and astrocytes) and recently identified CNS-resident immune cells, with a focus on their roles in development, health, and disease (Table 1).

**Table 1:** The major population of immune cells involved in neuroimmune interactions

Cell type	Location/Distribution	Specific markers/subtypes	Key functions	Ref
Microglia	CNS parenchyma	CD11b, F4/80, CX3CR1, IBA1, TREM119, P2RY12, Siglec-H	• Originate from the yolk sac during early development • Play key roles in immune surveillance and sensing danger signals • Engage in phagocytosis of neural progenitor cells and synaptic elements • Provide neurotrophic support through factors such as IGF-1 and BDNF • Respond to cytokines (e.g., IL-4, IFN-γ), enabling dynamic shifts in functional states	[67–89, 380]
Astrocytes	Throughout the CNS	GFAP, S100β, ALDH1L1	• Phagocytose apoptotic neurons and clear myelin debris • Provide structural support and regulate neurotransmitters to maintain extracellular homeostasis • Contribute to phagocytosis and overall debris clearance • Secrete IL-33 to recruit microglia for synaptic remodeling • Interact with vascular endothelial cells to help maintain BBB integrity	[92, 96, 97, 99, 104, 105]
Conventional T Cells	Dural/leptomeningeal regions, Choroid plexus, few in brain parenchyma	CD44, CD69, CD103	• Accumulate in the central nervous system (CNS) after birth • Modulate neuronal function through cytokine signaling (e.g., IFN-γ, IL-4) • Support the maturation and functional development of microglia	[10, 109–111, 114]
Regulatory T Cells (Tregs)	Dural/leptomeningeal regions, choroid plexus, and few in brain parenchyma	CTLA-4, ICOS, ST2,5-HT_7_	• Exhibit a tissue-resident phenotype, distinct from circulating Tregs • Contribute to inflammation resolution and tissue repair by producing IL-10, TGF-β, AREG, and SPP1, which suppress inflammation and promote recovery	[10, 113, 114, 159, 160]
Gamma Delta T Cells	Predominantly in the dural meninges	High IL-17A production	• Accumulate early in the meninges • Secrete high levels of IL-17A, modulating synaptic plasticity and influencing anxiety-like behaviors	[108, 112]
B Cells	Dural/leptomeningeal regions, choroid plexus, and few in brain parenchyma	Adults: Immature & B2 cells; Neonatal: B-1a cells	• Mediate local negative selection within the meninges • Promote proliferation of oligodendrocyte precursor cells during neonatal development	[120–123]
Border-Associated Macrophages (BAMs)	CNS borders: meninges, choroid plexus, and perivascular spaces	*Ms4a7, Ms4a6c, Tgfbi, Lyz2*	• Involved in antigen presentation and immune clearance • Regulate BBB permeability through NOX2.	[124–133, 179, 180]
Innate Lymphocytes (NK Cells & ILCs)	Dural/leptomeningeal regions, choroid plexus, and few in brain parenchyma	NK cells, ILC1s, ILC2s, few ILC3s	• Accumulate in the meninges after birth • ILC2 produce IL-13 to regulate inhibitory synapse development • ILC2 accumulation in the aged brain is associated with age-related cognitive function	[134–137]

### Microglia in health and homeostasis

Microglia are the primary resident immune cells of the CNS and play critical roles in its development and maintenance by continuously surveying the environment and responding to danger signals [67–74]. Microglia feature both common myeloid markers, such as CD11b, F4/80, CX3CR1, and IBA1 [75, 76],^,^ and microglia-specific markers, including TREM119, P2RY12, and Siglec-H [77–79]. Recent single-cell RNA-seq studies have revealed substantial transcriptional heterogeneity among microglia in homeostasis and disease [80–82]. For example, disease-associated microglia (DAMs) play a dual role in neurodegeneration, clearing toxic aggregates such as amyloid-beta (Aβ) in Alzheimer’s disease (AD) and sustaining chronic inflammation that contributes to neuronal damage [82]. During development, microglia expand rapidly to regulate neuronal fate through the phagocytosis of neural precursor cells [83]. They also secrete neurotrophic factors to support neurodevelopment and eliminate dysfunctional synapses through “find-me” and “eat-me” signals [84–86]. In the context of neuroimmune interactions, microglia can respond to peripheral immune changes by detecting cytokines such as IL-4 and IFN-γ, shaping their functional state accordingly [87]. Specifically, IL-4 promotes an anti-inflammatory phenotype by upregulating *Arg1* [88], whereas IFN-γ induces a proinflammatory state, leading to the release of TNF-α and IL-6 and the upregulation of MHC-II, which contributes to neuroinflammation [89].

### Astrocytes in health and homeostasis

Astrocytes are the most abundant glial cell type within the CNS; they exhibit context-dependent heterogeneity and respond dynamically to both physiological and pathological stimuli [90–94]. Astrocytes mediate neuroinflammation by acting as key intermediaries between the CNS and immune system, expressing cytokine receptors for IL-1α, IL-6, TNF-α, and IFN-γ, enabling them to sense immune signals [95].

During neurodevelopment, astrocytes share similar phagocytic capabilities as microglia do, clearing apoptotic neurons and myelin debris [96, 97]. In the absence of microglia, astrocytes enhance their phagocytic activity and release proinflammatory signals as a compensatory response [98]. The interaction between astrocytes and microglia is fundamental for CNS development. Specifically, astrocyte-derived IL-33 recruits and directs microglial synaptic pruning, a critical step in neural circuit maturation [99]. During development, astrocytes extend branched processes that directly ensheathe multiple synapses. This structural support is essential for proper synaptic excitability and the maintenance of conduction velocity [100–103]. Furthermore, the perivascular endfeet of astrocytes directly support BBB integrity [104, 105]. Even after the neurodevelopmental period, astrocytes continue to maintain synaptic integrity, neurotransmitter signaling, and water intake, all of which are necessary for healthy neural plasticity [106, 107]. Collectively, astrocytes contribute to CNS homeostasis through their roles in releasing neurotrophic factors, phagocytosing injured neurons, and maintaining a supportive environment necessary for optimal neural function.

### Brain-associated adaptive immunity in health

The CNS harbors resident adaptive immune cells, including specialized populations of T cells, which play vital roles in immune surveillance, neuroprotection, and inflammation regulation. In the dural meninges, γδ T cells accumulate from birth and persist lifelong, whereas the number of αβ T cells increases significantly after weaning [108, 109]. Meningeal T cells are composed mainly of CD44^+^CD62L^−^ memory T cells, with tissue-resident markers such as CD69 [108–110]. Early studies demonstrated cognitive impairments in T cell-deficient mouse models, which were reversed by repopulation with functional T cells [111]. These findings led to further investigations into the role of meningeal T cells in regulating brain function. For example, meningeal γδ T cells, which secrete high levels of IL-17A, play a crucial role in modulating anxiety-like behaviors by signaling to neurons with IL-17RA within the medial prefrontal cortex (mPFC) [112]. Tregs also exist in the meninges. A recent study revealed meningeal Tregs with Th1 and Tfh-like transcriptional signatures, which regulate adult hippocampal neurogenesis by controlling IFN-γ signaling [113].

In addition to the meninges, CD4^+^ conventional T cells (Tconvs) and Tregs are also present in mouse and human leptomeninges and the brain parenchyma [10, 114]. Approximately 2000 CD4^+^ T cells, including approximately 150 Tregs, have been identified in the brains of healthy mice, with brain-resident Tconvs featuring activation and residency markers such as CD44, CD69, and CD103 [10, 114]. Similarly, brain-resident Tregs are distinct from their circulating counterparts, exhibiting increased levels of the activation markers CTLA-4 and ICOS and the residency markers ST2 and CD69 [114]. Notably, the absence of brain-resident CD4^+^ T cells disrupts microglial maturation and synaptic pruning functions, resulting in abnormal behavioral phenotypes [114].

Recent research has demonstrated that distinct T cell subtypes differentially regulate neuronal function through cytokine-mediated mechanisms. Neurons and glial cells express receptors for T-cell-derived cytokines. For example, IL-4 receptor alpha (IL-4Rα), which binds to both IL-4 and IL-13, has been found on excitatory and inhibitory neurons of the hippocampus, and signaling along this axis is necessary for proper excitatory and inhibitory potentials in both humans and mice [115]. IL-4-deficient mice also exhibit a proinflammatory meningeal myeloid cell phenotype and cognitive deficits, which can be reversed by the transfer of T cells from wild-type mice, restoring cognitive function [116]. Additionally, GABAergic neurons carry IFN-γ receptor 1, linking IFN-γ to inhibitory signaling and cognitive behaviors [111, 117]. IL-17 has gained particular interest because of its capacity to serve as a neuronal signaling molecule and act beyond its traditional role as a proinflammatory cytokine. In *Caenorhabditis elegans*, IL-17 has been shown to directly bind to its receptor on neurons to increase synaptic activity and modulate behavior [118]. Accordingly, in mice, IL-17A and IL-17C can activate basolateral amygdala neurons with IL-17 receptor A, promoting anxiety-like behaviors, whereas IL-10 opposes this pathway. [119] Furthermore, IL-17A produced by meningeal γδ T cells modulates glutamatergic synaptic plasticity through glial BDNF production, influencing memory processes by balancing short- and long-term memory retention [108]. Collectively, these studies highlight the critical importance of brain-resident adaptive immune cells, particularly T cells, and their cytokine signals in CNS homeostasis, neuronal function, and cognitive and behavioral regulation.

B cells represent another critical adaptive immune cell type within the CNS. Constituting approximately 15–30% of CD45^high^ cells in the meninges, these B cells span diverse developmental stages, from pro-B cells to mature B cells [110, 120, 121]. While dura mater B cells, including immature B2 subsets, originate primarily from skull bone marrow, antigen-experienced B cells in the peripheral blood can infiltrate the meninges during aging [120]. Interestingly, autoreactive immature B cells that target CNS-specific antigens, such as myelin oligodendrocyte glycoprotein (MOG), undergo negative selection within the meninges, preventing their accumulation and potential autoimmune consequences [122].

B cells are also detected within neonatal brains, where their abundance peaks at P1 and remains elevated throughout early development [123]. Unlike predominantly immature dural B cells, neonatal brain B cells primarily consist of mature B-1a cells, whereas adult brain B cells are predominantly B-2 cells. The infiltration of neonatal B-1a cells is driven by the CXCL13–CXCR5 pathway, and these cells have been shown to promote oligodendrocyte precursor cell proliferation via IgM–Fcα/μR signaling [123]. This noncanonical role of B cells in the brain highlights their potential contribution to CNS regulation, suggesting a broader and previously underappreciated importance of B cells in brain function.

### Brain-associated innate immune cells in health and homeostasis

The CNS harbors distinct innate immune populations, notably BAMs and innate lymphocytes. BAMs constitute a diverse group of myeloid cells distributed across the meninges, choroid plexus, and perivascular spaces. Unlike parenchymal microglia, BAMs act as immune sentinels at CNS borders, interacting with both peripheral and CNS-resident cells. The majority of BAMs originate during developmental stages, with the exception of the choroid plexus and dural macrophages, which are replenished by monocytes in adulthood [124, 125]. These cells are characterized by the expression of macrophage-associated markers, such as CD206, CD169, CD163, CD36, and LYVE1, and exhibit a distinct transcriptional signature from parenchymal microglia, characterized by the expression of genes, including *Ms4a7*, *Ms4a6c*, *Tgfbi*, and *Lyz2* [126–128]. At key immune surveillance sites, such as the dural sinus and choroid plexus, MHC-II^high^ BAMs capture brain-derived antigens and engage in interactions with T cells [129, 130]. In the meninges, dural BAMs also communicate with other cells, including mural cells, to regulate antigen-dependent T cell responses [131]. By modulating vascular integrity, immune signaling, and BBB function, perivascular macrophages contribute to CNS homeostasis [132, 133].

Despite being one of the least studied lymphocyte populations in neuroimmune interactions, a few studies have reported the presence of natural killer (NK) cells and ILCs in CNS tissue under homeostatic conditions [110, 134, 135]. CNS-resident NK cells display distinct phenotypes from those of peripheral NK cells and are characterized by increased IL-2R and CD27 levels and a greater CD62L^+^ population, which is associated with NK maturity and enhanced cytotoxic function [110, 136]. The majority of these cells in the choroid plexus are NK/ILC1s and ILC2s, with only a few ILC3s being detected within the CNS [137]. A recent landmark study highlighted the critical role of meningeal ILC2-derived IL-13 in inhibitory synapse maturation and social behavior during early neurodevelopment through direct interactions with neuronal IL-13 receptors [135]. Furthermore, ILC2s accumulate in the choroid plexus of aged mouse brains, and intracerebroventricular transfer of activated ILC2s was found to rejuvenate the aged brain and enhance cognitive function in aged mice, highlighting the lifespan-regulating role of ILC2s in CNS health and aging [137].

### Neuroimmune interactions during disease

#### Microglia and astrocytes in neuroinflammation and disease

Glial cells harbor a dual role as regulators of neurodevelopment and homeostasis and drive neuroinflammation and neurological disorders. Both microglia and astrocytes are capable of producing proinflammatory cytokines, including IL-6, IL-1β, IL-23, IL-18, and TNF-α, and IL-1β, TNF-α, and IL-17A, respectively [138–140]. These interactions often form proinflammatory feedback loops. Specifically, microglia-derived IL-1α, TNF, and complement component 1q activate astrocytes via the NF-κB signaling pathway, resulting in reactive astrogliosis characterized by impaired neuronal support, reduced synaptic functions, and increased neuronal vulnerability [93]. This microglia‒astrocyte crosstalk is implicated in neurodegenerative diseases such as AD, Parkinson’s disease (PD), and Huntington’s disease (HD) [93]. Conversely, reactive astrocytes produce TNF-α and IL-6, further enhancing microglial activation and inflammation [141–144]. This glial-driven inflammation can drive neuronal dysfunction and degeneration. For example, TNF-α directly promotes neuronal death through the activation of caspase cascades, while IL-1β erodes synaptic sites by inducing pre- and post- synaptic dysfunction [145, 146]^.^ Additionally, the quantity of proinflammatory cytokines is directly correlated with the accumulation of Aβ plaques in AD, partly due to reduced Aβ clearance as well as increased astrocytic Aβ production [147, 148]. In addition to cytokines, glial cells are potent generators of reactive oxygen species (ROS) and NO, which further contribute to neurotoxicity and tissue damage [149]. Finally, chemokines produced by activated glial cells, such as CCL5, recruit peripheral immune populations, including T and B cells, to the brain [150].

In severe CNS injuries, such as ischemic stroke, microglia display fascinating heterogeneity in their function. Immediately following stroke, microglia take on an anti-inflammatory and tissue repair-associated phenotype, expressing CD206, *Arg1*, *Ccl22*, *Il10*, and *Tgfb1*, with enhanced phagocytic activity for clearing damaged tissue and supporting neuronal recovery and reinnervation [151]. However, approximately one week postinjury, a shift in the microglial phenotype occurs at the damage site, resulting in reduced phagocytosis and increased production of proinflammatory cytokines and chemokines, as well as the recruitment of other immune populations [151, 152]. Concurrently, ischemic stroke in Sprague–Dawley rats also induces pronounced reactive astrogliosis, characterized by elevated GFAP levels [153]. These reactive astrocytes are essential for reducing reactive oxygen species buildup following stroke and preventing further tissue damage [154]. Furthermore, astrocytes at injury sites are reprogrammed permanently to upregulate genes involved in wound healing, immune signaling, cell adhesion, border maturation, and microbial defense [155]. Astrocyte deficiency significantly disrupts vascular repair and remodeling, impairing motor function recovery after stroke and underscoring their crucial reparative role [156]. Together, microglia and astrocytes play dual roles: they are indispensable in maintaining CNS health, but are also pivotal contributors to neuroinflammation and the progression of neurological diseases.

#### Brain-resident adaptive immunity in tissue repair and neuroinflammation

During neurotraumatic disorders, T cells are essential mediators of tissue homeostasis, where they significantly influence neuroprotection and tissue repair [157, 158]. In ischemic stroke and traumatic brain injury, diverse immune populations, including T cells, infiltrate the CNS. Following brain injury, CD4⁺ T cells differentiate into various effector subsets, each of which secretes distinct cytokines that exert either proinflammatory or anti-inflammatory effects [157, 158]. In contrast, CD8⁺ T cells contribute to neuronal damage by releasing cytotoxic molecules, such as perforin and granzyme, which induce cell death and exacerbate neuroinflammation [157, 158]. In particular, the roles of Tregs in brain tissue repair are well established. Ito *et al*. demonstrated that ischemic stroke in mice leads to substantial accumulation of Tregs at the damage site, which is mediated by CCL1 and CCL20 [159]. These Tregs express unique signatures, such as the serotonin receptor 5-HT_7_, and are essential for producing amphiregulin to suppress neurotoxic astrogliosis [159]. Similarly, in a mouse model of neuromyelitis optica spectrum disorder, Tregs accumulate around lesion sites and, more specifically, colocalize with inflammatory microglia and macrophages [160]. These Tregs reduce the proinflammatory chemokines CCL1, CCL2, and CCL5, as well as the proinflammatory cytokines TNF-α and IFN-γ, through the production of IL-10 and TGF-β [160]. Earlier studies also support the notion of Treg–glial cell interactions, with one showing that Tregs are capable of restraining the LPS-induced inflammatory phenotypes of microglia and macrophages [161].

On the other hand, T cells can drive neuroinflammation. During multiple sclerosis (MS), autoreactive CD4⁺ T cells recognize myelin antigens, leading to CNS inflammation and demyelination [162]. B cells contribute by acting as antigen-presenting cells, sustaining T cell activation, and producing autoantibodies [163]. The interplay among these adaptive immune cells exacerbates neuroinflammation, leading to progressive neurodegeneration and functional impairment in MS.

#### Brain-resident T cells during neurological disorders

Adaptive immune cells play an active role in neurodegenerative diseases, such as AD. A recent study in a mouse model of tauopathy demonstrated that T cells infiltrate the brain, driven by an expanded population of CD8⁺ effector T cells [164]. Notably, depletion of either microglia or T cells significantly blocks tau-induced neurodegeneration [164]. Clinical studies have further noted lymphocyte infiltration in the brains of patients with AD and PD, highlighting adaptive immune infiltration as a conserved feature of neurodegenerative disorders and a potential target for therapeutic intervention [165, 166].

Adaptive immune cells may also drive neurodevelopmental disorders. T cell cytokines have been identified in the postmortem brain and serum of autism spectrum disorder (ASD) patients [167, 168]. Animal models, such as the maternal immune activation (MIA) model, have begun to reveal the neuroimmune mechanisms underlying neurodevelopmental disorders. MIA in pregnant mice is induced by LPS or poly(I:C), which are recognized by TLR3 and TLR4, respectively, and trigger pathogen-associated molecular patterns and damage-associated molecular patterns [169–171]. While the MIA model was initially linked to only IL-6, nearly a decade of research has revealed that a more robust immune response occurs within the MIA model [172]. In particular, MIA induced by poly(I:C) increases the serum levels of IL-6, TNF-α, and IL-1β in pregnant mice, but more importantly, IL-17A from Th17 cells is the key cause of MIA-associated behaviors [173, 174]. This mechanism is directly dependent on the presence of SFB in the maternal gut microbiota [174]. Gut microbial signals activate meningeal γδ T cells to produce IL-17A, resulting in IL-17A receptor signaling on neurons and subsequent abnormal anxiety-like behaviors [112]. Additionally, IL-17A secreted by meningeal T cells can activate border-associated macrophages, inducing ROS production and resulting in impaired cognitive function [175]. Interestingly, while IL-17A has been alluded to as a pathogenic cytokine in ASD, studies have also demonstrated a paradoxical capacity for IL-17A to restore neurotypical behaviors in MIA mice [173, 176]. To date, studies have demonstrated only insults from peripheral or meningeal T cells during neurodevelopmental disorders. It is yet to be determined whether parenchymal T cells also contribute to neuroinflammation. Taken together, these studies underscore the critical involvement of adaptive immune responses in diverse neurological conditions, revealing new avenues for therapeutic development targeting neuroimmune interactions.

#### Innate immune cells in neuroinflammation and disease

During neuroinflammation, BAMs act as early responders, rapidly initiating immune responses. Upon inflammatory activation, BAMs upregulate critical antigen-presenting molecules, such as MHC-II and CD44, to increase T-cell activation and contribute to the modulation of inflammatory processes [129, 177]. BAMs have also garnered significant attention in the context of neurodegenerative diseases, as they play essential roles in waste clearance, antigen recognition and presentation, and BBB regulation. For example, studies using the TgCRND8 AD mouse model have demonstrated that increased perivascular BAM turnover significantly promotes the clearance of Aβ peptides from cerebral vessels, implicating these macrophages in mitigating AD pathology [178]. Additionally, in a mouse model of PD characterized by alpha-synuclein (α-syn) accumulation, BAMs, rather than microglia, were identified as the key antigen-presenting cells responsible for mediating CD4⁺ T cell-driven neuroinflammation [179]. BAMs also regulate BBB permeability through the modulation of vascular oxidative stress, specifically by NADPH oxidase 2 (NOX2), highlighting their critical influence on vascular integrity and neuroimmune communication [180]. Collectively, these findings underscore BAMs as central regulators of CNS inflammation and homeostasis, coordinating peripheral immune signals and leukocyte trafficking during pathological states.

Innate lymphocytes similarly exert significant regulatory effects during neuroinflammation. In experimental autoimmune encephalomyelitis (EAE), NKp46⁺ ILCs are essential for initiating CNS infiltration via pathogenic CD4⁺ Th17 cells [181]. Specifically, genetic deletion of the transcription factor T-bet in NKp46⁺ ILCs significantly reduces Th17-mediated neuroinflammation, demonstrating their critical role in orchestrating adaptive immune responses [181]. Conversely, meningeal ILC2s exhibit neuroprotective properties, particularly following CNS injury, by upregulating calcitonin gene-related peptide (CGRP) and other neurotrophic molecules, thereby contributing to tissue repair and inflammation resolution [182]. These studies highlight innate lymphocytes as dynamic regulators of inflammatory and protective immune responses within the CNS, suggesting promising targets for therapeutic intervention in neuroinflammatory diseases.

### Signaling mechanisms from the gut to the brain

**Fig. 2:**
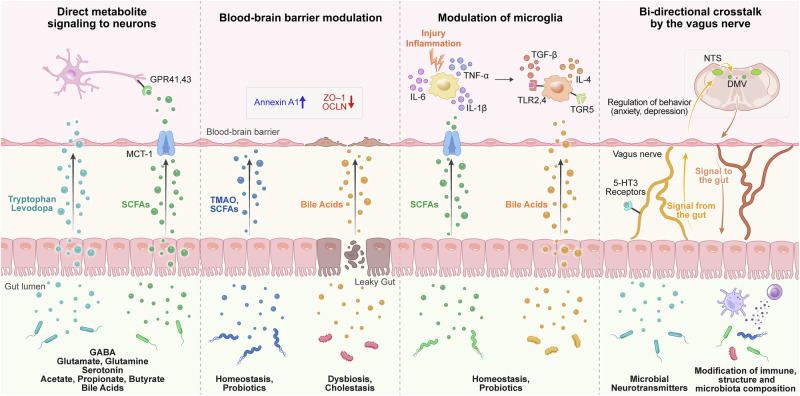
Gut-derived metabolites in the brain. Bidirectional crosstalk between the gut and brain occurs through numerous pathways. Gut microbiota-derived metabolites can directly affect neurons within the brain. While large metabolites cannot cross the BBB, precursors such as tryptophan and levodopa are capable of entering the brain. Additionally, BBB transporters, including MCT-1, can mediate the entry of SCFAs into the brain. The BBB itself can be affected by metabolites, as TMAO and SCFAs can enhance BBB integrity, whereas various bile acids can induce barrier disruption by modulating the expression of tight junction proteins. Once within the brain, SCFAs and bile acids can interact with microglia via TLR and TGR signaling, respectively, promoting an anti-inflammatory microglial phenotype, particularly under injury or inflammatory conditions. Finally, the vagus nerve acts as a major highway for gut‒brain crosstalk, facilitating bidirectional regulation. Gut-derived signals are transmitted via vagal afferents to the brainstem, where they are integrated and processed within interconnected nuclei, primarily the nucleus tractus solitarius (NTS) and the dorsal motor nucleus of the vagus (DMV). The NTS handles sensory input, whereas the DMV sends efferent signals that influence gut immunity and microbiota composition

With the rise of correlative studies linking the gut to the brain, the major question in the field is related to the mechanisms by which the gut can influence the brain. Numerous studies in recent decades have elucidated several distinct pathways by which the brain is linked with the brain (Fig. 2). Direct mechanisms include microbiota-derived metabolites that are taken up by neurons, whereas indirect mechanisms involve the activation of host immunity, which can influence neuroinflammation.

#### Neurotransmitters from the gut

The gut microbiota significantly influences the production and metabolism of major neurotransmitters, including dopamine, serotonin, glutamate, and gamma-aminobutyric acid (GABA). Specific microbes such as *Enterococcus faecalis* and *Enterococcus faecium* synthesize dopamine via levodopa decarboxylation, whereas serotonin synthesis in the gut is modulated primarily by species such as *Clostridium*, which influences enterochromaffin cells [183–185]. Additionally, *Lactobacillus* and *Bifidobacterium* species produce GABA through enzymatic glutamate decarboxylation [186]. However, interpreting the direct impact of gut-derived neurotransmitters on CNS function requires caution, as most peripheral neurotransmitters cannot freely cross the BBB under homeostatic conditions [187]. Nonetheless, several microbiota-derived precursor molecules, such as tryptophan and its metabolite 5-hydroxytryptophan (5-HTP), which are precursors to serotonin, as well as tyrosine and levodopa (L-DOPA), which are precursors to dopamine, can cross the BBB and subsequently modulate CNS neurotransmitter availability [187].

#### The role of short-chain fatty acids on neurons

SCFAs produced by commensal microbes are absorbed by colonocytes, enter portal circulation, and become an important source of energy for peripheral tissues, including the brain [188, 189]. SCFAs are capable of crossing the BBB through monocarboxylate transporter 1 (MCT-1), and their concentrations have been detected in human CSF and brain tissues [189–191]. Importantly, SCFAs regulate neuronal development and activity. For example, in human neural progenitor cell cultures, treatment with a mixture of acetate, propionate, and butyrate increased the expression of genes related to neurogenesis and proliferation while reducing the activity of apoptotic pathways [192]. In vivo, carbon-labeled acetate from fermentation in the gut was shown to transverse into the brain by crossing the BBB, upon which it is utilized by hypothalamic neurons for the generation of GABA and lactate [193]. Acetate can directly change the production of neuropeptides as well as hypothalamic AMP-activated protein kinase phosphorylation, ultimately regulating appetite behavior [193]. Similarly, butyrate can also suppress orexigenic neurons of the hypothalamus and neuron activity within the brainstem, reducing appetite and food intake in mice [194]. Appetite modulation in mice has also been demonstrated by SCFA receptors on vagal sensory neurons of the gut, particularly those for propionate [195].

In addition to feeding behaviors, SCFAs can control sympathetic responses to the gut microenvironment. The binding of propionate and acetate to GPR41 and GPR43 on the sympathetic superior cervical ganglion neurons of the spinal cord regulates calcium channel voltage and norepinephrine release, suggesting a mechanism of gastrointestinal sensing [196, 197]. Finally, GABA signaling in neurons may also be dependent on propionate. For example, in mice, propionate can enter GABAergic neurons and induce the accumulation of extracellular GABA and neuronal histone acetylation, a mechanism that may involve the inhibition of GABA transaminase [198].

Butyrate is among the best studied SCFAs because of its impact on neurons. Sodium butyrate, a histone deacetylase inhibitor, is capable of inducing long-term potentiation in the hippocampus, enhancing long-term memory [199]. In line with this finding, sodium butyrate is well documented to improve cognition and memory in several neurodegenerative disorder models by enhancing histone acetylation in the brain, including AD, HD, and PD [200–202]. In a mouse model of amyotrophic lateral sclerosis, phenylbutyrate inhibited programmed motor neuron apoptosis by acetylating NF-κB p50, resulting in elevated *Bcl-*2 expression and consequently blocking caspase activation [203]. Butyrate administration also provides neuroprotection following brain injuries, such as ischemic stroke. Sodium butyrate and moderate doses of acetate can reduce the area of tissue damage following middle cerebral artery occlusion (MCAO) by increasing GPR41, PI3K, and phosphorylated Akt levels and inhibiting neuronal apoptosis [204]. Moreover, butyrate supplementation was shown to improve angiogenesis following stroke in aged mice and improve BBB integrity by promoting IL-22 production by brain-associated ILCs and CD4^+^ T cells [205]. Specifically, IL-22 receptor activation on brain endothelial cells increases tight junction protein expression and endothelial cell proliferation, thus reducing inflammation-driven BBB permeability [205].

#### Short-chain fatty acids on brain-resident immune cells

Microglia exhibit significant phenotypic differences between GF and SPF mice. New studies have demonstrated that these differences may be due in part to commensal SCFAs. Mice lacking *Ffar2* (which encodes GPR43) for SCFAs exhibit microglial phenotypes similar to those of GF mice [206]. Feeding GF mice a mixture of SCFAs was sufficient to restore healthy microglial morphology and maturation signatures [206]. Specific SCFAs have the ability to modulate microglial function. Acetate restrains LPS-induced IL-1β, IL-6, and TNF-α production among microglia in vivo and in vitro through reversing NF-κB p65 phosphorylation while simultaneously increasing TGF-β and IL-4 production [207]. Acetate was also shown to have similar effects on primary astrocyte cultures by reversing MAPK p38 and NF-κB p65 phosphorylation [208].

Butyrate profoundly modulates microglial responses during neuroinflammation. During MCAO-induced ischemic stroke, sodium butyrate can induce histone modification in activated microglia to reduce the activity of proinflammatory genes while increasing the expression of genes involved in anti-inflammatory pathways [209]. Specifically, sodium butyrate can attenuate the LPS-induced expression of inflammatory *Tnf*, *Il6*, *Nos2*, and *Irf1* in microglia [209]. This may be due in part to TLR2 and TLR4 signaling, as they recognize microbial cell wall components and LPS. Notably, TLR2 and TLR4 have been identified on microglia and are implicated in microglial activation, phagocytic activity, and proinflammatory cytokine production [210–212]. TLR-mediated neuroinflammation is particularly common in neurodegenerative disorders such as AD, with microglial TLR2 and TLR4 recognition of HMGB1 or Tau exacerbating age-associated disorders [213, 214]. While the direct activation of microglial TLRs by microbial metabolites remains to be tested, neuraminidase from bacteria and viruses was shown to induce inflammatory phenotypes in microglia through TLR2 and TLR4 [215].

#### The role of bile acids in neurons

Bile acids represent another significant group of commensal-associated metabolites that impact CNS function. Gut microbes convert primary bile acids to secondary bile acids, which influence various neurological outcomes [2]. Tauroursodeoxycholic acid (TUDCA), a secondary bile acid, has demonstrated neuroprotective effects in 3-nitropropionic acid and R6/2 mouse models of HD [216, 217]. TUDCA has also been shown to ameliorate injury and improve motor function after stroke by reducing NF-κB and BCL-2 levels while enhancing the activity of the Akt- and Bad-mediated antiapoptotic pathways in rat neurons [218]. Moreover, TUDCA treatment ameliorates neurotransmitter imbalance and promotes hippocampal neuronal survival in a corticosterone-induced mouse model of depression [219].

However, bile acids also have neurotoxic potential. For example, during the early stages of AD, deoxycholic acid accumulates in the brain and can directly impair cognition by binding with the bile acid receptor Takeda G protein-coupled receptor 5 (TGR5) on excitatory neurons, leading to STAT3 phosphorylation, *Aph1* transcription, and increased Aβ production [220]. Interestingly, bile acids have also been linked with behaviors such as itching and scratching in mice [221]. During cholestasis, the stalling of bile acid transport and the accumulation of bile acids can activate TGR5 on dorsal root ganglia neurons, causing hyperexcitability and the production of gastrin-releasing peptide and leucine-enkephalin, which induce itch and analgesia [222]. Other behaviors, such as circadian rhythm sleep disorders during liver diseases, have been attributed to elevated bile acid levels within the suprachiasmatic nucleus and TGR5 binding to neurons [223]. These findings highlight bile acids as dynamic neuromodulators originating from the gut microbiota that are capable of both protective and detrimental effects on neuronal function.

#### Bile acids on brain-resident immune cells

Among neuroimmune cells, bile acids have beneficial and anti-inflammatory properties. For example, activated microglia express TGR5, which restricts neuroinflammation by stunting their activation, proliferation, and production of IL-1β, IL-6, and TNF-α [224]. This mechanism could be due to blockade of the NF-κB pathway, activation of nuclear factor-erythroid 2-related factor-2 (NRF2) signaling, and enhanced mitochondrial repair within activated microglia, which results in the suppression of the TNF-α positive feedback loop and CCL3 and CCL6 signaling [225, 226]. In this manner, ursodeoxycholic acid was shown to restrain inflammatory microglia during middle cerebral artery occlusion (MCAO) and improve neuron recovery and cognitive behavior in mice [227]. NF-κB inhibition by the binding of taurochenodeoxycholic acid to TGR5 has also been shown to drastically reduce inflammatory phenotypes among astrocytes during experimental autoimmune encephalomyelitis [228]. In the APP/PS1 mouse model of AD, TUDCA was able to restrain microglial and astrocyte activation and proinflammatory polarization and reduce Aβ generation, thus improving the rescue of cognitive deficits [229].

The dynamics of how bile acids impact the brain were recently characterized in depth. Profiling bile acids from various brain regions of young and aged mice has revealed a specific age-associated bile acid, tauro-β-muricholic acid (TβMCA), which can directly induce inflammatory microglia and subsequent neuroinflammation and cognitive decline [230]. Surprisingly, TβMCA accumulation and aging were correlated with a decline in gut microbes with bile salt hydrogenase, and these outcomes could be reversed by the introduction of young microbiota [230]. These dynamics indicate that bile acids should not be viewed as simply beneficial or harmful. However, each bile acid can trigger distinct responses in the brain and its various cell types. Neurons, endothelial cells, and glial cells may react differently to each bile acid, and altering the bile acid pool through the modulation of the gut microbiota could offer therapeutic potential.

#### Effects of commensal metabolites on BBB integrity

The gut microbiota profoundly influences BBB development and maintenance. GF mice display disrupted BBB integrity, characterized by reduced tight junction protein expression and increased permeability, underscoring the essential role of the microbiota in barrier homeostasis [231]. Various commensal-derived metabolites facilitate this crosstalk. Trimethylamine-*N*-oxide (TMAO) is the byproduct of dietary methylamine metabolism by commensal microbes from the *Anaerococcus, Clostridium, Escherichia, Proteus, Providencia*, and *Edwardsiella* genera [232]. Circulating TMAO enhances BBB integrity in both homeostasis and LPS-induced inflammation by increasing annexin A1 signaling in endothelial cells and protecting against inflammation-associated memory impairment in wild-type mice [233]. On the other hand, commensal metabolites can also be antagonistic to BBB integrity. For example, during cholestasis, elevated bile acid concentrations result in increased BBB permeability [234]. More specifically, chenodeoxycholic acid and deoxycholic acid directly reduce ZO-1 and OCLN tight junction proteins and disrupt the BBB [234]. Similar findings were reported in azoxymethane-induced mouse models of acute liver failure, in which serum bile acid levels increase, resulting in increased BBB permeability, increased bile acid concentrations in the brain, and neurological decline [235, 236].

An emerging hypothesis proposes a correlation between intestinal and BBB permeability. In one mouse model of sepsis-associated encephalopathy, disease onset is initiated by intestinal barrier disruption, which results in sepsis and neurological dysfunction [237]. Surprisingly, prophylactic treatment with a mixture of acetate, propionate, and butyrate in drinking water not only improved the intestinal barrier structure and integrity but also prevented BBB damage, neuroinflammation, and behavioral deficits [237]. Interestingly, sepsis reduces ZO-1 and OCLN levels in both the intestines and the brain, which SCFA treatment could rescue, suggesting a potential link between these two tissue barriers [237]. Similarly, colitis disrupts the intestinal barrier and results in gastrointestinal inflammation, which increases BBB permeability and neuroinflammation [238, 239]. A recent study using dextran sulfate sodium salt-induced colitis mice demonstrated that an enrichment in beneficial microbes, including *Lactobacillus, Akkermansia*, and *Faecalibaculum*, as well as elevated production of butyrate from the gut, and upregulated ZO-1 and OCLN levels in the BBB, enhancing its barrier function [240]. Finally, propionate has been shown to bind to GPR41 on BBB endothelial cells to maintain their integrity against nonspecific infections and oxidative stress [241]. Taken together, these findings emphasize that gut-derived microbial metabolites play critical roles in modulating BBB integrity, suggesting promising avenues for therapeutic intervention in neurological conditions associated with compromised barrier function.

#### Gut-activated peripheral immunity on the brain

**Fig. 3:**
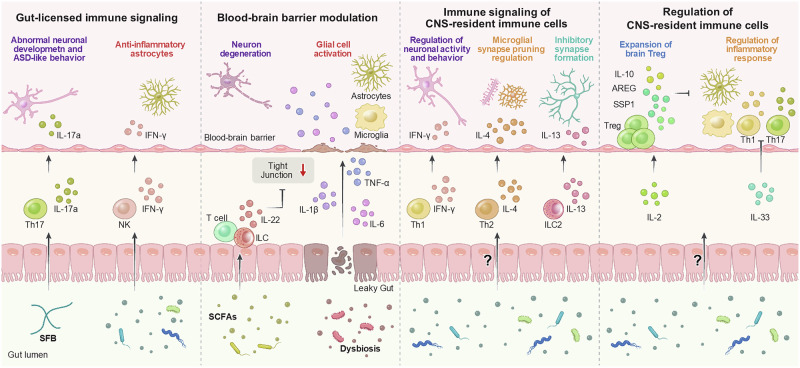
Key immune signals along the gut–immune–brain axis. Gut-mediated immune signaling regulates brain function. In the intestine, Th17 cells are induced by SFB and IL-17A during early brain development and are linked to ASD-associated behaviors. During inflammatory conditions such as EAE, IFN-γ-producing NK cells are recruited to the brain in a gut microbiome-dependent manner, where they promote anti-inflammatory astrocyte responses. Dysbiosis induces the production of proinflammatory cytokines that disrupt BBB integrity. Conversely, butyrate promotes IL-22 production by ILCs and CD4⁺ T cells, enhancing BBB stability via direct effects on endothelial cells. CNS-resident T cells secrete cytokines to modulate brain function: IFN-γ regulates neuronal activity, IL-4 regulates microglial synaptic pruning, and IL-4 and IL-13 aid in inhibitory synapse formation. Peripheral cytokines, including IL-2, which drives Treg expansion, and IL-33, which suppresses proinflammatory Th17/Th1 responses, also influence CNS-resident immune cells. The role of the gut microbiota in these processes remains to be elucidated

The gut microbiota and its metabolites may indirectly impact the brain through the modulation of peripheral immunity (Fig. 3). Gut-derived neurotransmitters impact not only neurons but also peripheral immune populations. Peripheral immune cells express various neurotransmitter receptors. For example, T cells have dopamine and serotonin receptors, which influence their proliferation, differentiation, cytokine secretion, and migration [242, 243]. Specifically, dopamine receptor activation can either enhance or suppress T cell activity, depending on the receptor subtype engaged [244]. GABA inhibits activation-induced calcium signaling and suppresses NF-κB activity in immune cells [245]. Similarly, glutamate receptor signaling enhances T cell migration, influencing T cell localization and function during immune challenges [246, 247]. Serotonin receptor signaling in T cells modulates their inflammatory responses, promoting regulatory T cell differentiation and immunosuppressive functions [248]. Notably, a recent study demonstrated that gut microbiota-derived serotonin directly signals to intestinal T cells, promoting Treg differentiation during neonatal development [249].

Gut-activated immune responses can influence the brain by transmitting inflammatory signaling molecules, such as cytokines, through the bloodstream. While the BBB typically restricts the direct entry of cytokine molecules into the brain, multiple studies have demonstrated the significant impact that peripheral cytokines can still have on brain function. IL-1β can signal through endothelial cells lining brain blood vessels, inducing the expression of *Cox2*, which contributes to sickness behavior and facilitates neutrophil infiltration into the brain [250]. Additionally, circulating proinflammatory cytokines such as TNF-α, IL-1β, and IL-6 can compromise BBB integrity by disrupting epithelial tight junctions, potentially allowing greater permeability to inflammatory molecules [251]. These circulating factors can elicit responses in CNS-resident immune cells. For example, in a house dust mite-induced allergic asthma mouse model, IL-4 can alter microglial phenotypes, resulting in reduced synaptic pruning capability and subsequently causing abnormal neural circuitry within the cerebellum [252]. Additionally, peripheral injection of IL-33 has been shown to mitigate EAE pathology in mice by shifting the Th17/Th1 balance toward a Th2 phenotype [253]. Similarly, the IL-2-mediated expansion of peripheral Tregs in humans and mice has demonstrated efficacy in various neurological disorder models, including amyotrophic lateral sclerosis [254]. Whether circulating cytokines, particularly those influenced by the gut microbiota, can modulate brain-resident immune cells under homeostatic conditions remains an open question and warrants further investigation.

A recent study demonstrated interorgan immune communication, showing that gut-derived immune cells migrate to distant tissues [255]. Gut-educated immune cells can directly migrate into the CNS, enabling them to interact with brain-resident cells. For example, a distinct subset of astrocytes characterized by LAMP1 and TRAIL1 (LAMP1^+^ TRAIL^+^ astrocytes) plays a critical role in limiting CNS inflammation in EAE mice [256]. During homeostasis, the development of these astrocytes relies on IFN-γ produced by meningeal NK cells [256]. Using photoconvertible Kaede mice, researchers revealed that these IFN-γ-producing meningeal NK cells originate from the gut, with the microbiota directly modulating IFN-γ production [256].

Significant alterations in CNS-resident immune cells often follow changes in the gut microbiota. For example, meningeal IgA-secreting plasma cells are notably reduced in GF mice but can be restored upon gut microbiota recolonization [257]. Similarly, in GF mice, brain-resident conventional CD4⁺ T cells undergo significant expansion following colonization with the wild-type microbiota, indicating that peripheral T cell activation by the microbiota promotes CNS infiltration [114]. Once in the CNS, these peripherally activated CD4⁺ T cells support the maturation and functional regulation of brain-resident microglia [114]. The gut also clearly contributes immune cells to pathological conditions that modulate neuroinflammation, as demonstrated by the migration of gut-derived IgA^+^ plasma cells into the CNS during EAE and the infiltration of intestinal T cells and monocytes into the CNS in a mouse model of stroke [258, 259]. Collectively, these findings underscore the gut as a critical reservoir and regulator of peripheral immune cells that populate and influence the CNS.

### Signaling mechanisms from the brain to the gut

English phrases such as ‘butterflies in your stomach’ during moments of anxiety and nervousness or ‘gut-wrenching feelings’ during upsetting moments allude to rudimentary ideas that the brain may influence our gut. Since ancient times, stress has often been implicated in gastrointestinal distress, including heartburn, constipation, and diarrhea. Today, emerging studies are beginning to identify several mechanisms by which signals from the brain act on the gastrointestinal tract to regulate digestion, the gut microbiota composition, the microbially derived metabolite pool, and gastrointestinal immunity.

#### Bidirectional crosstalk through the vagus nerve: Gut-to-brain

The vagus nerve is the tenth cranial nerve that runs from the brainstem down into the abdomen, innervating muscular and mucosal tissues of the gastrointestinal tract [260]. The vagus nerve regulates the parasympathetic control of the digestive tract, as well as the delivery of sensory information from the gut to the brain. The vagus nerve is suspected to be one mechanism by which the gut microbiota regulates the brain. For example, vagus nerve activity and its ability to stimulate brainstem neurons are dependent on the presence of the gut microbiota and its metabolites [261]. Additionally, in mice treated with antibiotics, vagotomy is capable of reducing antibiotic-induced anxiety and depression [262, 263]. Similarly, vagotomy suppresses LPS-induced inflammation-mediated depression in C57BL/6 mice by reducing synaptic activity in the brain and suppressing proinflammatory cytokine levels [264]. A similar protective effect of vagotomy was also shown in *Lactobacillus* and IL-6-induced mouse depression models, suggesting that the vagus nerve plays a crucial role in gut-immune-brain crosstalk [265]. Interestingly, bacteria from the same genus have different neurological impacts through the vagus nerve. For example, while *L. instinalis* and *L. reuteri* strains induce depression via the vagus nerve, *L. rhamnosus* JB-1 is capable of reducing stress-associated anxiety and depression in mice by regulating synaptic activity within the gut and the brain [265–267]. These differences might be attributed to metabolic regulation by the vagus nerve.

Serotonin receptors (specifically the 5-HT3 receptor) are present within the vagus nerve, and serotonin itself can activate these neurons [268]. A recent study demonstrated that the gut microbiota of stress-induced mice activates the vagus nerve to elicit serotonin and dopamine dysregulation in the brainstem and hippocampus and depressive behaviors [269]. Additionally, it was recently proposed that even microbial extracellular vesicles can reduce serotonin and BDNF signaling by translocating into the brain through the vagus nerve, causing depression and neuroinflammation [270]. Vagotomy can ameliorate these effects by promoting neurogenesis and suppressing TNF-α, IL-6, and IL-1β in the hippocampus [269, 270]. Furthermore, vagotomy can abrogate other metabolic and neurotransmitter signals from the gut to the brain, including SCFAs, BDNF, and GABA, demonstrating that the vagus nerve is required for gut-to-brain metabolic signaling [261, 266, 271, 272].

#### Bidirectional crosstalk through the vagus nerve: Brain-to-gut

While the vagus nerve is largely known for its role in mediating gut-to-brain signals, the brain also has the potential to regulate the gut although the vagus nerve. Early reports demonstrated that vagus nerve stimulation can activate STAT3 signaling in intestinal macrophages and suppress intestinal inflammation [273]. More recently, an all-encompassing murine study elegantly illustrated a broader understanding of how the CNS senses peripheral immune activation through the vagus nerve and, in turn, can modulate immune homeostasis [274]. Briefly, neurons in the brainstem are activated by peripheral immune insult through the vagus nerve, as vagotomy is capable of eliminating brainstem activation [274]. Surprisingly, two separate vagal neurons were identified, one with the capacity to respond to proinflammatory cytokines to induce a negative feedback response and the other that can respond to anti-inflammatory cytokines to boost a positive feedback loop [274]. All of these responses are dependent on GABAergic neuron activation within the brainstem to suppress LPS-mediated inflammation [274].

In addition to immune modulation, the vagus nerve can directly control the gastrointestinal tract and the gut microbiota. For example, the activation of the dorsal motor nucleus in vagus neurons increases fat absorption and weight gain [275]. This mechanism is attributed to GABA receptor activity among vagal neurons, which regulates jejunal microvillus length and reduces the surface area by which fat can be absorbed [275]. This capacity to alter the intestinal structure also implies changes in the gut microbiota. Indeed, in the loperamide and restraint-stress model for murine irritable bowel syndrome (IBS), vagotomy improved IBS symptoms by increasing defecation, fecal water content, and constipation levels [276]. These changes were accompanied by shifts in the microbiota composition and microbial activity [276]. Similar observations were made in an MCAO-induced ischemic stroke model, in which vagotomy ameliorated both intestinal and BBB damage by suppressing mast cell activation in these regions [277]. Vagotomy in mice subjected to MCAO altered the gut microbiota composition by increasing the Firmicutes/Bacteroidetes ratio; altering the composition of the flora; reducing LPS endotoxin levels in the serum, brain, and colon; and suppressing the serum levels of TNF-α, IL-6, and IL-1β [277]. Taken together, the vagus nerve is a bidirectional pathway in which gut–immune–brain crosstalk is transmitted and regulated.

#### The effects of the hypothalamic‒pituitary‒adrenal axis on the regulation of the gut

Stress is colloquially thought to weaken the immune system, which is scientifically evidenced by cancer studies demonstrating stress-induced T cell suppression and tumor growth [278–280]. The hypothalamic‒pituitary‒adrenal (HPA) axis is a neuroendocrine system that regulates stress responses via hormone feedback loops between the hypothalamus, pituitary gland, and adrenal gland [281]. Briefly, stress triggers the release of corticotrophin-releasing hormone (CRH) from the hypothalamus, which causes the pituitary glands to secrete adrenocorticotrophic hormone (ACTH). ACTH travels to the adrenal glands, where it stimulates cortisol production. Cortisol acts as a negative feedback loop to suppress hypothalamus and pituitary gland activity. Various cell types throughout the gastrointestinal tract express cortisol receptors, suggesting that cortisol and the stress response may act directly on the regulation of the gut and its microbiota [282].

Among patients with infantile epileptic spasms syndrome (IESS), a form of drug-resistant epilepsy syndrome, the serum levels of IL-2, IL-4, IL-6, and IL-17a are elevated, along with CRH and the abundance of *Sutterellaceae* and *Sutterella* in the gut, indicating immunological, neuroendocrine, and gut microbiota disturbances in this neurological disorder [283]. Surprisingly, treatment with ACTH not only reduces CRH, ACTH, and cortisol but also lowers cytokine levels in the serum [283]. The stress response, particularly the levels of ACTH and cortisol, are well documented for their role in systemic immune regulation. ACTH therapy can lower lymphocyte counts in the serum, resulting in greater suppression of Th cells than of cytotoxic T cells [284]. In fact, in vitro, ACTH has the opposite effect on cytotoxic T cells by enhancing memory T cell cytotoxic responses and IFN-γ production [285].

Cortisol also has immunosuppressive effects, particularly among innate immune populations, and reduces TNF-α and IL-6 levels [286]. In particular, clinical studies on cortisol levels following exercise or circadian rhythm also suggest that elevated serum cortisol levels are correlated with suppressed lymphocyte presence and proinflammatory cytokine levels [287, 288]. Traumatic brain injuries can elicit a similar response in wild-type mice, causing elevated plasma cortisol levels and decreased circulating T cell populations, suggesting that the brain can regulate immune responses through the HPA axis [289]. These systemic immune modulations may directly impact gastrointestinal immune homeostasis, although more research is necessary in this regard.

The changes to the immune system caused by the HPA axis may involve crosstalk between host immunity and the gut microbiota. In IESS patients, ACTH treatment decreases *Lachnospiraceae incertae sedis* abundances while increasing *Alistipes* and *Rikenellaceae abundances* [283]. Following ACTH therapy, the changes in *Alistipes* and *Rikenellaceae* are directly correlated with IL-17a, IFN-γ, IL-6 and IFN-α levels, whereas *Sutterellaceae* is positively correlated with CRH levels and negatively correlated with IL-2 and TNF-α [283].

The HPA axis may also mediate maternal stress in infants, altering their microbiome. In a clinical study of maternal stress and depression, both prenatal and postnatal maternal stress directly regulated cortisol levels in mothers and correlated with infant microbiota composition [290]. In particular, high maternal stress and depression during pregnancy result in lower infant microbial diversity, lower *Lachnospiraceae*, and higher *Enterobacteriaceae* abundances [290]. On the other hand, maternal postpartum stress and depression reduce the abundance of *Bifidobacterium and Lachnospiraceae* and increase the abundance of *Streptococcaceae* in infants, presumably due to the effects of breastfeeding [290]. Importantly, some of these infant microbiota changes are correlated with maternal and infant cortisol levels during these periods, with elevated maternal cortisol during pregnancy correlating with lower *Bacteroides* in infants and increasing the abundance of the genus postpartum [290]. This study reveals an interesting dynamic in how maternal stress, both during pregnancy and after birth, affects infant microbiota composition through the HPA axis.

#### Neurotransmitters that regulate gut permeability and microbes

The enteric nervous system (ENS) regulates digestion and gastrointestinal activity through the secretion of various neurotransmitters. Epithelial cells of the intestinal lining and smooth muscles throughout the gut express receptors for neurotransmitters, allowing the modulation of barrier integrity, peristalsis, and lumen maintenance [291]. For example, acetylcholine induces smooth muscle contractions necessary for intestinal motility and the progression of digestion [292]. The gut microbiota is correlated with intestinal motility, with the enrichment of certain microbes, such as *Rumioncoccaceae, Eubacterium, Butyricimonas*, and *Bacteroidetes*, during constipation and the depletion of *Prevotella, Faecalibacterium*, and *Dialister* during diarrhea [293, 294]. This is also evidenced by observations that changes in intestinal motility and the gut microbiota are key factors in IBS, along with inflammatory immune activation, all three factors that converge in a feedback loop [295].

ENS neurotransmitters are also tied directly to the regulation of intestinal barrier functions, which control both the gut microbiota composition and immune responses. Acetylcholine has been well studied for its ability to regulate mucus levels in the intestinal lumen by inducing mucus secretion in goblet cells, regulating microbial populations [296–298]. One mechanism may involve the regulation of stress responses against intestinal epithelial cells, as acetylcholine has been shown to block mitochondrial unfolded protein responses in *C. elegans* [299]. In a mouse model of DSS-induced colitis, acetylcholine receptors on epithelial cells enhanced protection against colitis progression by increasing the expression of tight junction proteins and inhibiting NF-κB [300]. This mechanism is in part due to the ability of acetylcholine to negate TNF-α signaling in epithelial cells, thus demonstrating its immunomodulatory potential [300]. Overall, acetylcholine is a key mediator of gastrointestinal disorders.

Serotonin is capable of reducing ileal inflammation in mice by inducing acetylcholine release from myenteric plexus neurons, which bind to acetylcholine receptors on macrophages to reduce infiltration [301]. Furthermore, in *C. elegans*, microbial infections can stimulate acetylcholine production in neurons, which activates Wnt signaling and the production of antimicrobial peptides in gastrointestinal epithelial cells [302]. The gut microbiota is recognized to directly produce neurotransmitters that can affect intestinal function. For example, in *Drosophila melanogaster*, *L. plantarum* was found to produce acetylcholine, which directly altered gut motility [303]. These findings suggest bidirectional communication through neurotransmitter signaling, in which both the brain and commensal microbes can regulate the gastrointestinal tract as well as responses to gastrointestinal inflammation.

### Evidence for a gut‒immune‒brain axis in neurological disorders

To date, this review has addressed evidence for gut microbiota-mediated regulation of host immunity, the roles of brain-associated immune cells in health and disease, and various pathways by which commensals influence and are influenced by the brain. While very few studies have comprehensively combined all three aspects in a single model, we synthesized available studies to hypothesize that the gut microbiota can mediate neurological disorders through the activation of host immunity. In this section, we discuss the current evidence highlighting how the gut microbiota influences neurodevelopmental and neurodegenerative disorders by modulating host immune pathways (Table 2).

**Table 2:** Major clinical and clinically relevant studies have revealed gut‒brain or gut‒immune‒brain axes in neurological disorders

Disorder	Model	Axis	Key findings	Ref.
ASD	Humanized GF and BTBR Mouse Models	Gut Microbiota-Brain	Human ASD patient microbiota can induce ASD phenotypes in GF mice ↑ Bacteriodetes, β-Proteobacteria, Lactobacillales, Clostridiacseae, and Enterobacteriaceae in ASD-FMT murine offspring 5-aminovaleric acid and taurine improves ASD	[381]
	Clinical	Gut Microbiota-Brain	↑ *Clostridium, Streptococcus, Acinetobacter*, and *Alcaligenaceae* in ASD ↑ Arginase metabolism in ASD	[305]
	Clinical	Gut Microbiota-Brain	↓ Butyrate producing microbes in ASD	[306]
	Clinical	Gut Microbiota-Brain	↓ *Bacteroidetes* ↑ *Catenibacterium*, *Tenericutes* in ASD	[307]
	Clinical	Gut Microbiota-Brain	↑*Lactobacillaceae, Bacteroides, Parabacteroides, Proteus* ↓ *Bifidobacterium, Prevotella, Bacteroidetes, Blautia* in ASD ↑ Zinc, Copper, Nickel in ASD	[308]
	Clinical	Gut Microbiota-Metabolite-Brain	↓*Prevotella, Megamonas* ↑ *Escherichia-Shigella, Dialister, Bifidobacterium* Disrupted lipid, vitamin, glycan, xenobiotic, and amino acid metabolisms	[325]
	Clinical	Metabolite-Brain	↑ Glutamate, citrulline, acetylcarnitine, lactate, choline, ornithine, glycine, histidine, free fatty acids in ASD	[310–313]
	Clinical	Metabolite-Brain	↑ GABA degradation into butyrate	[314]
	Clinical	Metabolite-Brain	↑ lactate, alanine, glycerol-3-phosphate, threonine, linoleic acid, linoleylcarnitine, cholesterol, ceramides ↓ anti-inflammatory and antioxidant molecules (glutathione, carnosine, carnitine, betaine, 5’methyltetrahydrofolic acid, CoQ10), bile acid metabolism, dopamine, serotonin in ASD	[315]
	Clinical	Immune-Brain	↑ IL-1β, IL-6, IL-8, IL-12, IL-17, IFN-γ, and TNF-α in blood among ASD	[316–318]
	Clinical	Immune-Brain	↑ IL-1β, IL-6, IL-17, and TNF-α in brain among ASD	[319]
AD	Clinical	Gut Microbiota-Brain	↓ Microbiota richness, Firmicutes, *SMB53, Dialister, Clostridium, Turicibacter, Bifidobacterium, Adlercreutzia* ↑ Bacteroidetes, *Blautia, Phascolarctobacterium, Gemella, Bacteroides, Alistipes*	[327]
	Clinical	Gut Microbiota-Brain	↑ *Dorea formicigenerans*, *Oscilibacter* sp. 57_20, *Faecalibacterium prausnitzii, Coprococcus catus, Anaerostipes hadras* in preclinical AD ↑ *Bacteroides caccae, Bifidobacterium longum, Bacteroides faecis, Bacteroides salyersiae, Bacteroides massiliensis* in healthy	[382]
	Clinical	Gut Microbiota-Brain	↑ Correlation between higher cognitive performance with *Bacteroides massiliensis, Bifidobacterium pseudocatenulatum, Fusicatenibacter saccharivorans, Eggerthella lenta*	[330]
	Clinical	Gut Microbiota-Immune-Brain	↓ Butyrate-producing bacteria ↑ proinflammatory-associated bacteria in AD	[329]
Depression	Clinical	Gut Microbiota-Immune-Brain	↑ *Paramecium excretum, Parasutterella*, lysine biosynthesis and methionine biosynthesis pathways in depression Correlation with CD8, CD11b, and CD27 B cells in depression	[335]
	Clinical and Humanized Mouse Model	Gut Microbiota-Immune-Brain	↑ *Bacteroides*, TNF, MCSF, IL-12 ↓ *Clostridiumn, Roseburia, Haemophilus, SMB53*, and *Turicibacter* FMT of depression microbiota can induce inflammatory depression via TLR4/NF-κB SCFA can regulate inflammation and symptoms	[336]
	Clinical and Chronic Social-defeat Stress Mouse Model	Gut Microbiota-Immune-Brain	↑ *Lactobacillus* correlated with lower depression rating scales in depression patients ↑ γδ17 T cells in colon and meninges in depressed mice	[337]

#### The gut–immune brain axis in neurodevelopmental disorders

Numerous microbiota profiling studies from clinical and preclinical settings have identified alterations in microbial compositions among ASD patients, suggesting gut microbial signatures as potential biomarkers. A meta-analysis of 18 studies encompassing 493 ASD patients and 404 neurotypical individuals revealed distinct gut microbiota patterns in ASD, characterized by elevated levels of *Bacteroides*, *Parabacteroides*, *Clostridium*, *Faecalibacterium*, and *Phascolarctobacterium* and reduced *Coprococcus* and *Bifidobacterium* abundances [304]. In a cohort of ASD patients in China, *Clostridium, Streptococcus, Acinetobacter*, and *Alcaligenaceae* were enriched in ASD patients, along with arginase metabolism, which has been proposed as a biomarker for ASD [305]. A separate ASD cohort from China also revealed fewer butyrate-producing bacteria, including *Faecalibacterium* and *Coproccus*, among ASD patients and disrupted SCFA metabolism and neurotoxin degradation pathways, which were suggested to be ASD biomarkers [306].

Interestingly, geographic variations significantly influence microbiota profiles. A study conducted among Lebanese ASD patients and their neurotypical siblings revealed lower *Bacteroidetes* and higher *Catenibacterium* and *Tenericutes abundances* in ASD patients, whereas a separate study in Saudi Arabia reported higher *Bacteroides* and lower *Bifidobacterium* and *Prevotella abundances* in ASD patients than in their siblings [307, 308]. Such drastic differences in microbiota compositions across geography highlight the challenges in using gut microbiota compositions as a sole diagnostic biomarker. While cross-cohort studies do exist, such as the one comparing the Shenzhen and Moscow ASD cohorts, the vast differences in diets, culture, and other lifestyle factors result in underpowered analyses, with the example study identifying only two species, *Eubacterium_sp_CAG_248* and *P. copri*, as shared ASD-associated biomarkers [309]. Thus, a more overarching approach, one that takes into consideration the underlying mechanistic pathways, is needed.

Metabolomic studies provide deeper insight into the functional impact of the gut microbiota during ASD pathogenesis. Metabolite analyses have revealed increased blood levels of glutamate, citrulline, acetylcarnitine, lactate, choline, ornithine, glycine, histidine, and free fatty acids among ASD patients [310–313]. Additionally, metagenomic analysis of ASD patients from the United States of America revealed that dysfunctional dopamine, GABA, and serotonin pathways are correlated with specific enzymes and oral microbes that may be responsible for these effects [314]. For example, GABA degradation into butyrate was enriched in ASD, which is mediated by 4-aminobutyrate transaminase, glutamate dehydrogenase, and ButCoA acetyl transferase, and these enzymes were correlated with the abundances of *Actinomyces*, *Cardiobacterium*, and *Streptococcus* species among ASD patients [314].

A recent longitudinal metabolomic study comparing newborn and 5-year-old ASD patients revealed that the majority of the metabolic differences in ASD patients were accounted for by 14 key biochemical pathways [315]. In this study, metabolic signatures were nearly identical between pre-ASD newborns and neurotypical newborns, and differential signatures were observed only in 5-year-olds, suggesting that ASD-associated metabolic signatures develop with age and the maturation of their respective gut microbiota [315]. In pre-ASD newborns, several sphingolipid metabolic pathways and chenodeoxyglycocholic and taurodeoxycholic bile acids are increased, whereas serotonin, dopamine, niacin, vitamin B3, and flavin adenine dinucleotide are decreased [315]. Among 5-year-olds, ASD was correlated with lower sphingomyelin, bile salt, tryptophan, serotonin, vitamin, and L-carnitine metabolic pathways [315]. Taken together, ASD metabolic signatures are correlated with decreased anti-inflammatory and antioxidant responses, with decreased glutathione, carnosine, 5’-methyltetrahydrofolic acid, and CoQ10 levels, whereas the levels of metabolites involved in stress response pathways, such as lactate, glycerol, cholesterol, and ceramides, are increased [315].

Immunological phenotypes, particularly with respect to patients’ gut microbiota and metabolic composition, may also prove to be robust biomarkers. Inflammation is a hallmark of ASD, with elevated levels of IL-1β, IL-6, IL-8, IL-12, IL-17, IFN-γ, and TNF-α in plasma and serum samples [316–318]. IL-1β, IL-6, IL-17, and TNF-α are also elevated in the postmortem brains of ASD patients [319, 320]. Interestingly, a correlative analysis between metabolites altered in ASD patients revealed that glutamine, serine, proline, histidine, lysine, taurine, 5-aminolevulinic acid, and 2-hydroxyglutaric acid, among others, are involved in inflammatory pathways [321]. For example, glutamine, glycine, and taurine all have anti-inflammatory and antioxidant effects [322–324]. An imbalance in such metabolites during ASD may be causative for enhanced neuroinflammation. With this concept, meta-analyses are necessary to link microbiota compositions with metabolic pools and identify pathways involved in the regulation of inflammation. Studies applying this approach are slowly emerging, with one metagenomic study linking the abundance of *Ruminococcus* in individuals with ASD with increased middle chain fatty acid production, which is known to induce proinflammatory IL-12 [325]. Additionally, the present study revealed lower *Prevotella* abundance among constipated ASD patients, a genus that has often decreased across ASD studies [308, 325]. *Prevotella* are important producers of succinic acid, which has anti-inflammatory effects by suppressing IL-6, TNF-α, IL-1β, and nitric oxide production in macrophages, further supporting a microbe‒metabolite‒immune link in ASD [325, 326].

#### The gut–immune–brain axis in neurodegenerative disorders

Compared with healthy individuals, AD patients have a reduced richness in the gut microbiota composition, with lower Firmicutes and higher Bacteroidetes [327]. According to previous studies, *Clostridiaceae*, *Dialister*, *Clostridium*, *Erysipelotrichaceae*, *Lachnoclostridium*, and *Subdoligranulum* are decreased in AD patients, whereas *Blautia, Phascolarctobacterium, Gemella, Bacteroides, Alistipes, Odoribacter*, and *Barnesiella* are increased [327–329]. In particular, elevated genera such as *Bacteroides* and *Blautia* are positively correlated with higher CSF concentrations of tau and Aβ, whereas less abundant genera, including *Dialister*, are negatively correlated with these genera [327]. SCFA-producing bacteria, including *B. massiliensis, B. pseudocatenulatum*, and *Fusicatenibacter saccharivorans*, are also indicative of better cognitive function among elderly individuals with mild cognitive impairment [330]. It was hypothesized that *B. pseudocatenulatum*, a producer of acetate, could participate in anti-inflammatory signaling and the maintenance of intestinal immune homeostasis [330, 331]. Notably, the microbiota of AD patients can impair neurogenesis in the hippocampus as well as cognitive and memory capacity in adult male Sprague‒Dawley rats [332]. Furthermore, the human AD gut microbiota promotes an intestinal inflammatory state by lowering P-glycoprotein expression and slightly elevating MRP2 protein levels in cultured intestinal epithelial cells, thus modulating inflammatory responses against commensals [329].

Human primary brain cells cultured with *B. fragilis-*derived LPS drive NF-κB, which may help promote neuroinflammation and pathogenesis during AD [333]. Interestingly, in a GF APPPS1 mouse model of AD, a reduced Aβ load was directly associated with diminished levels of SCFAs in the serum, and supplementation with an acetate, butyrate, and propionate mixture was capable of doubling Aβ plaque accumulation within the brain [334]. SCFAs were shown to enhance microglial localization to Aβ plaques; however, SCFAs also reduced microglial uptake of Aβ and upregulated APOE, suggesting an immunosuppressive function of SCFAs in the context of AD [334]. While neuroinflammation is considered a pathogenic hallmark of AD, few studies have directly connected the microbial and metabolic signatures of AD with their immunomodulatory capacities. Future research in AD will need to address whether the gut microbiota and its metabolites are capable of modulating neuroinflammation and thereby affecting the outcome of AD pathology.

#### The gut–immune brain axis in psychiatric disorders

In addition to neurodevelopmental and neurodegenerative disorders, the interplay of the gut-immune-brain axis is also observable in neuropsychiatric disorders. A genome-wide association study among depression patients revealed significant correlations between gut microbes and their predicted metabolic pathways, immune dysfunction, and incidence of depression [335]. Specifically, *Paramecium excretum* and *Parasutterella* were identified as having causal associations with depression, along with the lysine biosynthesis and methionine biosynthesis pathways [335]. CD8^+^ T cells, CD11b^+^ monocytic myeloid-derived suppressor cells, and CD27^+^ B cells are also correlated with depression, with CD27^+^ B cells specifically mediating the effects of the microbial methionine biosynthesis III pathway during disorders [335].

Depression patients also present increased TNF, macrophage colony-stimulating factor (MCSF), and IL-12 in the intestinal mucosa and reduced abundances of *Clostridium, Roseburia, Haemophilus, SMB53*, and *Turicibacter*, which are positively correlated with propionate and butyrate levels [336]. Interestingly, the gut microbiota of patients with treatment-resistant inflammatory depression differed from that of patients with other types of depression in that inflammation-associated *Bacteroides* were enriched, whereas SCFA-producing *Clostridium* were decreased [336]. This was consistent with reduced butanoate metabolism; increased TLR-4, NF-κB, NLRP3, Caspase-1, TNF, and MCSF; and decreased ZO-1 and OCLN levels in the intestinal mucosa [336]. Notably, these phenotypes could be recapitulated in wild-type mice that received fecal microbiota transplantation (FMT) of inflammatory depression patients’ feces, and *C. butyricum* treatment could ameliorate depression phenotypes by reducing inflammation, demonstrating a causal effect [336]. Furthermore, in a chronic social defeat stress mouse model of depression, inhibiting intestinal γδ T cells with TCR γ and δ antibodies could directly prevent stress-induced γδ T-cell accumulation in the meninges and subsequent behavioral deficits [337]. This evidence highlights both the immunomodulatory function of the gut microbiota and the capacity for immune modulation to directly affect depressive disorders, supporting the theory of a gut–immune–brain mechanism in neuropsychiatric disorders.

### Targeting the gut–immune–brain axis for therapeutics

The gut–immune–brain axis has emerged as a crucial target for developing novel therapies for neurological disorders, with increasing evidence underscoring its role in neuroinflammation, immune regulation, and brain function. Therapeutic strategies aimed at this axis focus primarily on two approaches: immune-based interventions that modulate inflammatory pathways and microbiota-based therapeutics (MBTs) that leverage the gut microbiome to influence brain health. Understanding how immune dysfunction and microbial imbalances contribute to neurological conditions provides valuable insights for precision medicine. In this section, we explore current strategies targeting immune and MBTs and their potential to restore homeostasis within the gut–immune–brain axis, ultimately improving neurological outcomes (Table 3).

**Table 3:** Clinical and animal models for testing various immunological and gut microbiota-based therapeutics

Disorder	Model	Axis	Therapeutic	Mechanism	Ref.
Stroke, MS	Clinical	Immune	Fingolimod	Lymphocyte sequestration in the lymph nodes	[340]
Stroke	Clinical	Immune	Anakinra	IL-1R antagonist	[341]
	Clinical	Immune	IL-2 AAV vector	Treg expansion	[343]
AD	Clinical	Immune	Aducanumab	N-terminal Aβ-targeting antibody	[346]
	Clinical	Immune	Lecanemab	Aβ protofibrils-targeting monoclonal antibody	[348]
	Clinical	Immune	Donanemab	N-terminal truncated Aβ-targeting IgG	[347]
	Clinical	Immune	Masitinib	Mast cell & microglia-targetting tyrosine kinase inhibitor	[349]
	Clinical	Immune	PD-1/PD-L1 Blockade	PD-1 checkpoint blockade leads to IFN-γ-mediated recruitment of macrophages for Aβ clearance	[350]
	APP/PS1 Mouse	Gut-Immune-Brain	*L. paracasei* D3.5, *L. rhamnosus* D4.4, *L. plantarum* D6.2, *L. rhamnosus* D7.5 and *L. plantarum* D13.4, *E. raffinosus* D24.1, *E. INBio* D24.2, *E. avium* D25.1, *E. avium* D25.2 and *E. avium* D26.1	↑ BBB and intestinal barrier integrity ↓ Microglial activation, IL-6, TNF-α	[370]
	3xTg-AD Mouse	Gut-Immune-Brain	*Lactobacillus plantarum* KY1032 and *Lactobacillus curvatus* HY7601	↓ Microglia and astrocyte density ↑ Neuron density	[371]
PD	Clinical	Immune	Sargramostim	GM-CSF-induced DC enhancement for Treg induction	[353]
	Clinical	Immune	Azathioprine	Immunosuppressant that suppress lymphocyte proliferation	[354]
	Clinical	Immune	AKST4290	G protein-coupled C-C chemokine receptor type 3 antagonist	[355]
	Clinical	Gut-Brain	*L. paracasei* DG and inulin fiber	↑ Cognitive function, improved bowel function, altered microbiota composition	[372]
	Clinical	Gut-Immune-Brain	*L. rhamnosus, E. faecium, L. acidophilus, L. plantarum*	↓ TNF-α and IL-6 in plasma	[374]
	MPTP-Mouse	Gut-Immune-Brain	*L. rhamnosus* E9	Improve motor function by reducing ROS in brain and enhancing intestinal barrier integrity	[373]
Epilepsy	Clinical	Immune	Tocilizumab	IL-6 receptor inhibitor	[358]
	Clinical	Immune	Anakinra	Anti-IL-1 receptor antagonist	[359]
	Clinical	Immune	Natalizumab	Anti-α4-integrin antibody	[360]
ASD	BTBR Mouse	Gut-Brain	*C. butyricum*	Enhancement of intestinal barrier function via SCFA and HDAC1 modulation	[365]
	PPA- Rat	Gut-Immune-Brain	*L. rhamnosus* and luteolin	↓ TNF-α and IL-6 ↑ GABA, glutathione, glutathione peroxidase in brain	[366]
	PPA-Rat	Gut-Immune-Brain	*B. longum* BB536	↑ Oxytocin ↓ IFN-γ	[367]
	MIA Mouse	Gut-Immune-Brain	*P. goldsteinii* MTS01	↓ Colonic TNF-α, IL-6, IL-1β ↑ Antioxidant pathways and ↓ glutamate receptor signaling in brain	[368]
Depression	Chronic Stress Mouse	Gut-Immune-Brain	*L. plantarum ATCC 793 (Lp793), B. longum ATCC 15707 (Bl15707)*, and botanical derived polyphenolic preparation	↑ CTLA4^+^ Tregs ↓ Th17 in gut, IL-1β, IL-17a in serum, IL-1β in cortex	[376]
Depression and Anxiety	LPS Mouse	Gut-Immune-Brain	*L. lactis, L. cremoris, L. diacetylactis, L. acidophilus*, and lactic yeasts	↓ IL-12, TNF-α, IL-1β	[377]
ADHD, ASD, Emotion Regulation	Clinical	Gut-Immune-Brain	Synbiotic 2000	↓ IL-12/IL-23p40, soluble intercellular adhesion molecule 1 ↑ Propionic Acid in blood	[378]

#### Immune therapies for neurological disorders

Currently, there are no FDA-approved immunomodulatory therapies for stroke. Despite promising results from medical and physical interventions in experimental stroke models, successful translation into clinical applications remains a challenge [338, 339]. Among potential candidates, fingolimod, a sphingosine-1-phosphate receptor (S1P) modulator that prevents lymphocyte egress from the lymph nodes, has shown encouraging outcomes in preclinical and early clinical studies [340]. Additionally, anakinra, an IL-1 receptor antagonist, has demonstrated potential therapeutic efficacy in several clinical trials [341]. Recent preclinical studies have identified Tregs as promising therapeutic targets for stroke. The expansion of Tregs via IL-2/IL-2 antibody complexes and brain-targeted IL-2 overexpression in astrocytes via AAV vectors have shown beneficial effects in MCAO and cortical impact mouse models of stroke [342, 343]. The modulatory effects of Treg expansion may be mediated through the regulation of glial cell activation, facilitated by Treg-derived molecules such as SPP1 and AREG [159, 344].

Immune-based therapies for AD primarily target Aβ [345]. In particular, immunization with Aβ peptides and the administration of anti-Aβ antibodies have emerged as key therapeutic strategies for AD. Notably, two monoclonal antibodies, aducanumab and lecanemab, have received traditional FDA approval for the treatment of AD [346–348]. Furthermore, masitinib, a tyrosine kinase inhibitor, has demonstrated neuroprotective effects through the inhibition of mast cells and microglia/macrophage activity, and clinical trials have shown significant cognitive improvement [349]. Additionally, PD-1/PD-L1 immune checkpoint inhibitors are currently under clinical investigation for AD. Preclinical studies have shown that PD-1/PD-L1 blockade can mobilize monocyte-derived macrophages, enabling their infiltration into the brain to reduce pathology and combat cognitive decline in 5XFAD AD mice [350, 351]. An antibody targeting PD-L1 has been developed to stimulate the immune system by inhibiting immune checkpoint proteins, and this approach is currently in early-phase clinical trials for AD.

The aggregation of α-syn into Lewy bodies is a pathological hallmark of PD. Both passive and active immunization against α-syn aggregates are being explored in ongoing trials [352]. Additional immune-targeted strategies include the use of Sargramostim, a human recombinant granulocyte‒macrophage colony-stimulating factor that increases the number of Tregs, which can modestly improve motor functions [353]. Furthermore, the immunosuppressant azathioprine has been reported to improve cognition in PD patients by inhibiting DNA synthesis [354]. Moreover, AKST4290, a chemokine inhibitor that targets C-C chemokine receptor 3 to reduce immune cell infiltration, is currently being evaluated as a potential PD treatment [355].

In addition to neurotraumatic and neurodegenerative diseases, neurodevelopmental disorders, such as pediatric epilepsies, are increasingly recognized as targets for immunotherapy, particularly in cases where immune dysfunction contributes to seizure pathology. In IESS and Lennox–Gastaut syndrome (LGS), ACTH and steroids have demonstrated therapeutic efficacy through immune suppression [356, 357]. Additionally, cytokine-targeting therapies, including tocilizumab (an anti-IL-6 receptor inhibitor) and anakinra (an anti-IL-1 receptor antagonist), have been shown to be effective in treating immune-mediated epileptic syndromes, such as new-onset refractory status epilepticus (NORSE) and febrile infection-related epilepsy syndrome (FIRES) [358, 359]. Natalizumab, an anti-α4-integrin antibody that blocks leukocyte migration to the brain, is also currently under clinical investigation for drug-resistant epilepsy, with some successful cases reported in Rasmussen’s encephalitis [360, 361]. These advancements underscore the potential of immunotherapies as promising treatment strategies for pediatric and drug-resistant epilepsies.

Finally, ASD represents another promising area for immune-based intervention. Clinical observations have revealed reduced frequencies of Tregs in individuals with ASD [362, 363]. Accordingly, therapeutic strategies aimed at selectively expanding Tregs through IL-2/anti-IL-2 complex therapies are currently being explored as potential approaches to alleviate ASD-associated inflammation and symptoms [364].

#### Microbiota-based therapeutics for neurological disorders

MBTs come in multiple forms, including probiotic live bacteria, prebiotics, synbiotics, postbiotics, and microbial metabolites [1]. In recent years, numerous reviews and meta-analyses have characterized clinical and animal studies testing the efficacy of MBTs in various neurodevelopmental, neurodegenerative, and neuropsychiatric disorders [2]. However, studies that address all three aspects of the gut–immune–brain axis simultaneously remain limited. In the following section, we highlight the latest studies in which MBTs are utilized specifically for their immunomodulatory potential in the context of neurological disorders.

MBTs have been extensively studied in ASD, where inflammation and microbial dysbiosis are key pathological features. For example, the BTBR and valproic acid mouse models for ASD are characterized by systemic inflammation and weakened barrier functions [365]. In these mice, treatment with SCFA-producing *C. butyricum* improved intestinal barrier integrity by increasing the levels of TREK1, CLDN1, CNDN3, and OCLN tight junction proteins and reducing the levels of IL-6, TNF-α, and IFN-γ in the colon, resulting in reduced ASD-associated behaviors [365]. Similar findings have also been reported in a rat model of propionic acid-induced ASD, in which treatment with a mixture of *L. rhamnosus* and luteolin (an antioxidant ingredient of artichokes) reduces proinflammatory TNF-α and IL-6 levels and elevates GABA, glutathione, and glutathione peroxidase levels in the brain [366]. Additionally, *B. longum* BB536 mirrors these effects in propionic acid-induced ASD rats by reducing IFN-γ levels within the brain [367]. In the MIA mouse model of ASD, *P. goldsteinii* MTS01 ameliorated antisocial and anxiety-like behaviors by reducing colonic TNF-α, IL-6, and IL-1β in the intestines while increasing the activity of antioxidant pathways and decreasing glutamate receptor signaling in the hippocampus [368]. Finally, prebiotics, such as 3% galacto-oligosaccharide/fructo-oligosaccharide, have been shown to prevent valproic acid-induced ASD-associated social and cognitive deficits by suppressing microglial activation, lowering Th1 and Th17 and increasing Treg populations, improving intestinal barrier function, and normalizing the gut microbiota composition and SCFA pool [369].

MBTs are also being explored for the treatment of neurodegenerative diseases, such as AD and PD. In an APP/PS1 mouse model for AD, a mixture of *L. paracasei* D3.5, *L. rhamnosus* D4.4, *L. plantarum* D6.2, *L. rhamnosus* D7.5, *L. plantarum* D13.4, *E. raffinosus* D24.1, *E. INBio* D24.2, *E. avium* D25.1, *E. avium* D25.2 and *E. avium* D26.1 improved cognitive function by enhancing BBB and intestinal barrier integrity, reducing activated microglia, and reducing IL-6 and TNF-α within the brain, plasma, and intestines [370]. This study reveals a clear link between barrier function and immune modulation across the intestines, periphery, and brain. Furthermore, in female 3xTg-AD mice, a mixture of *Lactobacillus plantarum* KY1032 and *Lactobacillus curvatus* HY7601 is capable of improving memory by reducing microglia and astrocyte densities while increasing neuron populations [371]. MBTs have also demonstrated therapeutic capacity in PD. A synbiotic combination of *L. paracasei* DG and inulin fiber improved cognitive function among PD patients [372]. This mechanism could be immune-modulated, as other *Lactobacillus* strains, such as *L. rhamnosus* E9, are capable of reducing PD-associated inflammation by improving intestinal barrier integrity [373]. Furthermore, a consortium of *L. rhamnosus, E. faecium, L. acidophilus, and L. plantarum* can significantly reduce plasma IL-6 and TNF-α levels in PD patients [374]. Similar results were found in in vitro cultures of peripheral blood mononuclear cells from PD patients, which showed suppressed inflammatory cytokine and ROS production when these cells were treated with six individual probiotic strains, supporting a probiotic–immune link in PD [375].

Neuropsychiatric disorders may also be targeted through MBTs. In a chronic unpredictable stress model of murine depression, a synbiotic combination of *L. plantarum* ATCC 793 (Lp793)*, B. longum* ATCC 15707 (Bl15707), and a botanical-derived polyphenolic preparation (BDPP) promoted CTLA4^+^ Treg expansion, lowered Th17 cells in the gut, lowered IL-1β and IL-17a levels in the serum, and lowered prefrontal cortex IL-1β levels, which attenuated depression and anxiety-like behaviors [376]. Another probiotic mixture containing *L. lactis, L. cremoris, L. diacetylactis, L. acidophilus*, and lactic yeasts also confers protection against LPS-induced depression and anxiety in CD1 mice by reducing the serum levels of IL-12, TNF-α, IL-1β, and TNF-α in the brain [377]. In a clinical trial for ADHD, Synbiotic 2000, a mixture of *Pediococcus pentosaceus* 5-33:3/16:1, *L. casei subsp*. paracasei F19, *L. plantarum* 2362 and beta-glucan, inulin, pectin and resistant starch fibers, was capable of lowering neuroinflammatory IL-12/IL-23p40 and soluble intercellular adhesion molecule 1 while increasing propionic acid levels in the blood [378]. Notably, while Synbiotic 2000 did not improve ADHD symptoms compared with placebo, Synbiotic 2000 showed efficacy in improving autism symptoms in children and emotion regulation among adults [379]. Collectively, these studies suggest the potential of MBTs as immunomodulatory therapeutics for neurological conditions. However, many studies are still merely correlative. Future investigations should explicitly test causality by systematically inhibiting individual components of the gut–immune–brain axis to establish direct therapeutic mechanisms.

## Conclusion and future directions

The intricate interplay among the gut microbiota, immune system, and CNS has emerged as a compelling area of research, offering new perspectives on pathogenesis and potential treatment strategies for neurological disorders. The evidence discussed throughout this review highlights not only how the gut microbiota and its metabolites shape immune and neurological homeostasis but also how immune and neural signals reciprocally modulate microbial communities.

In neurological disorders, increasing amounts of data highlight correlations between disease progression, gut microbial dysbiosis, altered metabolite pools, and inflammation. This leads to the hypothesis that neurological disorders may be induced by a gut-immune mechanism. Aberrations in the gut microbial composition can lead to dysregulated immune signaling, increased neuroinflammation, and disruptions in blood‒brain barrier integrity, all of which can exacerbate neurological dysfunction. This growing body of evidence underscores the therapeutic potential of targeting the gut microbiota and immune pathways to develop novel interventions for neurological diseases.

To achieve this, several critical directions must be pursued. One promising approach is the identification of new biomarkers. Microbial metabolites and immune signatures have potential for predicting disease onset, disease progression, and therapeutic response in several neurological disorders. Validating and clinically applying these biomarkers could significantly improve diagnostic precision and enable personalized treatment strategies. With respect to therapeutic targets, current findings suggest that the compromised BBB, a hallmark of various neurological disorders, may be effectively addressed through novel interventions. These could include barrier-enhancing microbiota-derived metabolites or probiotics to restore BBB integrity. Additionally, targeting microbial metabolites and immune factors may also be effective in treating neurotraumatic diseases by modulating local immune responses and promoting neuronal recovery. Another crucial consideration is the interindividual variability in microbiome composition and immune profiles. Patient-specific interventions, such as personalized dietary strategies, CRISPR–Cas9–based microbiome editing, and tailored probiotic formulations, are essential for effective treatment. Alongside these clinical efforts, mechanistic studies must continue to unravel the complex interactions within the axis.

Promoting interdisciplinary collaboration among immunologists, neuroscientists, microbiologists, bioengineers, and clinicians will be crucial for driving innovative, integrative discoveries. As research in this field continues to evolve, the gut–immune–brain axis is likely to redefine our understanding of neurological disorders, paving the way for innovative microbiome-based and immune-modulating therapies.
